# Prognostic significance of CD56 expression in newly diagnosed multiple myeloma and its correlation with clinicopathological features

**DOI:** 10.3389/fonc.2026.1855388

**Published:** 2026-08-17

**Authors:** Pei Pei, Jiaxin Lv, Boyuan Zheng, Kangqi Lv, Junjun Bai, Lin Li, Qinglin Song, Zhizhong Lu, Han Xu, Yi Zhang

**Affiliations:** 1 Laboratory of Hematological Diseases, Jiaozuo People’s Hospital Affiliated to Henan Medical University, Jiaozuo, Henan, China; 2 Department of Hematology, Jiaozuo People’s Hospital Affiliated to Henan Medical University, Jiaozuo, Henan, China; 3 School of Clinical Medicine, Henan University, Kaifeng, China; 4 Department of Clinical Laboratory, Jiaozuo People’s Hospital Affiliated to Henan Medical University, Jiaozuo, Henan, China; 5 School of Basic Medical Sciences & School of Public Health, Faculty of Medicine, Yangzhou University, Yangzhou, Jiangsu, China; 6 Department of Clinical Pharmacy, Jiaozuo People’s Hospital Affiliated to Henan Medical University, Jiaozuo, Henan, China

**Keywords:** bortezomib, CD56, newly diagnosed multiple myeloma, overall survival, progression-free survival

## Abstract

**Objective:**

To investigate the correlation of CD56 expression with clinical prognosis and adverse prognostic indicators in patients with newly diagnosed multiple myeloma (NDMM).

**Methods:**

We conducted a retrospective cohort study of 153 NDMM patients treated at Jiaozuo People’s Hospital Affiliated to Henan Medical University between May 2014 and August 2024. Patients were stratified into the bortezomib cohort and the non-bortezomib cohort based on their treatment regimens, and were further divided into CD56^+^ group and CD56^−^ group according to CD56 expression status determined by flow cytometry. Prognosis and clinicopathological characteristics were compared between groups stratified by CD56 expression and treatment regimen.

**Results:**

No statistically significant differences in median progression-free survival (PFS) or overall survival (OS) were observed between the CD56^+^ and CD56^−^ groups. Median PFS and OS were significantly longer in the bortezomib-based cohort than in the non-bortezomib cohort, whereas no significant differences were found among patients treated with VCD, VTD, or VRD regimens. Within the bortezomib-based cohort, no significant difference in median PFS or OS was observed between CD56^+^ and CD56^−^ patients. In contrast, within the non-bortezomib cohort, CD56^−^ patients had significantly shorter median PFS and OS than CD56^+^ patients. Among CD56^−^ patients, no significant differences in median PFS or OS were observed across the VCD, VTD, and VRD regimens. CD56 deficiency was associated with multiple adverse prognostic indicators, including thrombocytopenia, elevated β_2_-microglobulin (β_2_-MG), bone marrow fibrosis, advanced International Staging System (ISS) stage, complex karyotype, and cytogenetic aberrations.

**Conclusion:**

Bortezomib-based therapy may attenuate the adverse prognostic impact associated with CD56 deficiency. CD56 expression has prognostic significance in NDMM and is closely correlated with multiple adverse clinicopathological indicators; however, it does not serve as an independent prognostic factor for NDMM patients.

## 1 Introduction

Multiple myeloma (MM) is a malignant proliferative disorder originating from bone marrow plasma cells and is characterized by profound cytogenetic and biological heterogeneity, with substantial variability in clinical manifestations and survival outcomes among patients (1, 2). In recent years, the clinical application of proteasome inhibitors, immunomodulatory drugs, and novel targeted agents has significantly improved remission rates and long-term survival in patients with MM (3, 4). However, the disease remains incurable, and most patients eventually experience relapse, drug resistance, or disease progression (5). Therefore, identifying reliable biological markers and optimizing prognostic stratification systems are clinically important for guiding individualized therapy and improving outcomes in high-risk patients.

Marked heterogeneity in MM underlies variable treatment responses and challenges in prognostic stratification (6, 7). This heterogeneity manifests in chromosomal aberrations, dysregulated gene expression, epigenetic dysregulation, an abnormal tumor microenvironment, and altered immunophenotypes (8). Currently, the International Staging System (ISS) and Revised International Staging System (R-ISS) rely primarily on serum biochemical parameters and cytogenetic abnormalities for risk stratification, without fully incorporating immunophenotypic features of plasma cells. Consequently, they cannot fully capture the complex biological heterogeneity of MM, especially the diverse antigen expression profiles on clonal plasma cells (9–11). CD56 (neural cell adhesion molecule), a key transmembrane adhesion molecule on myeloma cells, is weakly expressed or absent on normal plasma cells but positive in the majority of patients with NDMM (12). It serves as a core immunophenotypic marker for distinguishing malignant from benign plasma cells by flow cytometry.

Functionally, CD56 mediates adhesive interactions between myeloma cells and the bone marrow stroma and extracellular matrix, thereby regulating tumor cell homing, proliferation, migration, and invasiveness, and ultimately influencing disease aggressiveness (13). In the conventional chemotherapy era, lack of CD56 expression has been strongly associated with advanced ISS stage, bone marrow fibrosis, thrombocytopenia, elevated β_2_-microglobulin, TP53 deletion, and complex karyotypes, emerging as a potential adverse prognostic marker (14, 15). In the novel-agent era, the prognostic significance of CD56 remains controversial. Several studies report that CD56 negativity portends inferior survival, whereas accumulating real-world evidence indicates that bortezomib-based therapy may attenuate the adverse prognostic impact of CD56 negativity, suggesting its prognostic effect is treatment-dependent (15–17).

Existing studies on CD56 are mostly limited by small, single-center cohorts and lack stratified validation across different treatment regimens. Accordingly, this retrospective study enrolled 153 patients with NDMM treated between May 2021 and August 2024. We systematically analyzed the correlation between CD56 expression and progression-free survival (PFS) and overall survival (OS), and explored its associations with clinicopathological parameters, bone marrow fibrosis, and cytogenetic abnormalities. We further evaluated whether bortezomib-based therapy could reverse the adverse prognostic effect of CD56 negativity. This study aims to clarify the prognostic value of CD56 in the novel-agent era and provide real-world evidence for refining risk stratification and guiding individualized management in NDMM.

## 2 Materials and methods

### 2.1 Patient data

We performed a retrospective analysis of 153 patients with NDMM admitted to Jiaozuo People’s Hospital Affiliated to Henan Medical University between May 2014 and August 2024. All patients met the diagnostic criteria specified in the *Guidelines for the diagnosis and management of multiple myeloma in China* (2024 Revision) (18). Demographic, clinical, and laboratory data were collected from electronic medical records.

### 2.2 Immunophenotyping

2–3 mL of bone marrow aspirate was collected in EDTA anticoagulant tubes at diagnosis before any treatment and processed within 4 hours. Immunophenotypic analysis was performed using multi-color flow cytometry. The antibody panel included: CD38-FITC (cat. no. 665004; BD Biosciences), CD138-APC (cat. no. 130-117-395; Miltenyi Biotec), CD56-BV421 (cat. no. 664148; BD Biosciences), and CD45-V500-C (cat. no. 662912; BD Biosciences). Additional markers (CD19, CD27, CD81, CD117) were also evaluated but are not the focus of this study. Red blood cells were lysed using BD FACS Lysing Solution (BD Biosciences). Data acquisition was performed on a BD FACSCanto II flow cytometer (BD Biosciences), and at least 100,000 events were acquired per tube. Plasma cells were identified as CD38^+^/CD138^+^ cells with high side scatter properties. CD56 expression was assessed on the gated plasma cell population. A threshold of ≥20% positive cells was used to define CD56 positivity. Isotype matched control antibodies were used to set the cut-off for positivity.

### 2.3 Treatment regimens

Patients were stratified into the bortezomib-based cohort and non-bortezomib-based cohort according to their treatment regimens. The bortezomib-based cohort received bortezomib-containing combination regimens, including VTD (bortezomib + thalidomide + dexamethasone), VRD (bortezomib + lenalidomide + dexamethasone), VCD (bortezomib + cyclophosphamide + dexamethasone), and VD (bortezomib + dexamethasone). The non-bortezomib-based cohort was treated with VAD (vincristine + doxorubicin + dexamethasone) or MP (melphalan + prednisone) regimens. Drug dosages were adjusted appropriately in the event of grade 3–4 adverse events during treatment.

### 2.4 Response assessment

The overall response rate (ORR) was defined as the sum of complete response (CR), very good partial response (VGPR), and partial response (PR) rates. Response evaluation was performed according to the International Myeloma Working Group (IMWG) consensus guidelines (19).

### 2.5 Follow-up

The follow-up period ended on August 28, 2025. Progression-free survival (PFS) was defined as the time from diagnosis to disease progression, relapse, or all-cause death. Overall survival (OS) was defined as the time from diagnosis to the last follow-up or all-cause death.

### 2.6 Statistical analysis

We performed data analyses using SPSS software (version 22.0). Categorical data are presented as counts (percentages), and group comparisons were conducted using the chi-square test or Fisher’s exact test, as appropriate. Survival analyses were performed using the Kaplan–Meier method, with univariate survival analysis conducted via the log-rank test. Multivariate survival analysis was performed using the Cox proportional hazards regression model. A two-sided *P* < 0.05 was considered statistically significant.

## 3 Results

### 3.1 Baseline characteristics of the study cohort

A total of 153 NDMM patients were included in this study. The cohort comprised 85 males (55.6%) and 68 females (44.4%), with a median age of 62 years (range, 37–83 years). Disease subtypes were distributed as follows: IgG (64 cases, 41.8%), IgA (43 cases, 28.1%), IgD (3 cases, 2.0%), light-chain (38 cases, 24.8%), and non-secretory (5 cases, 3.3%). Bone marrow FISH was performed in 90 patients (58.8%), detecting IgH rearrangement in 45 cases (50%), TP53 deletion in 11 cases (12.2%), RB1 deletion in 40 cases (44.4%), and 1q amplification in 48 cases (53.3%). Circulating plasma cells (CPCs) were detected in 14.3% (18/126) of the patients. Among the 153 NDMM patients, the proportion of CD56-positive plasma cells ranged from 0.3% to 91.4%, with a median of 30.7%. Additional laboratory findings and clinicopathological characteristics are summarized in **Table 1**.

**Table 1:** Baseline characteristics of patients with NDMM.

Characteristics	Data
Age (years), median (range)	62 (37–83)
Sex [*n* (%)]	
Male	85 (55.6)
Female	68 (44.4)
M-protein type [*n* (%)]	
IgG	64 (41.8)
IgA	43 (28.1)
Others	46 (30.1)
DS stage [*n* (%)]	
I+II	33 (21.6)
III	120 (78.4)
ISS stage [*n* (%)]	
I+II	73 (47.7)
III	80 (52.3)
R-ISS stage [*n* (%)]	
I+II	69 (59.5)
III	47 (40.5)
BMPC (%), median (range)	30.1 (0–98.5)
Hb (g/L), median (range)	88 (30–149)
PLT (×10^9^/L), median (range)	167 (11–433)
ALB (g/L), median (range)	33 (15–49)
Cr (μmol/L), median (range)	176.8 (21–935.3)
Ca (mg/L), median (range)	2.4 (1.6–3.7)
LDH (U/L), median (range)	202 (48–1703)
β_2_-MG (mg/L), median (range)	7 (1.5–43)

### 3.2 Univariate analysis of prognostic factors for PFS and OS

Patients with ISS stage III disease, R-ISS stage III disease, extramedullary disease (EMD), positive CPCs, myelofibrosis (MF) grade 2, TP53 deletion, 1q amplification, platelet count ≤100×10^9^/L, serum calcium >2.75 mg/L, lactate dehydrogenase (LDH) >200 U/L, β_2_-microglobulin (β_2_-MG) >5.5 mg/L or the overall response rate exhibited significantly shorter PFS (all *P* < 0.05). Similarly, patients with R-ISS stage III disease, EMD, positive CPCs, complex karyotype, IgH rearrangement, TP53 deletion, platelet count ≤100×10^9^/L, serum calcium >2.75 mg/L, LDH >200 U/L, β_2_-MG >5.5 mg/L or the overall response rate had significantly shorter OS (all *P* < 0.05) (**Table 2**).

**Table 2:** Univariate analysis of prognostic factors in 153 patients with NDMM.

Parameters			n	PFS		OS	
				*χ* *^2^*	*P*	*χ* *^2^*	*P*
Sex		Male	85	0.096	0.757	1.361	0.243
		Female	68				
Age (years)		≤65	99	0.108	0.742	0.572	0.450
		>65	54				
DS stage		I+II	33	1.000	0.317	1.228	0.268
		III	120				
ISS stage		I+II	73	5.992	0.014	2.570	0.109
		III	80				
R-ISS stage		I+II	69	5.851	0.016	5.590	0.018
		III	47				
M-protein type		IgG	64	4.929	0.085	0.965	0.617
		IgA	43				
		Others	46				
Extramedullary disease		Yes	30	7.596	0.006	16.811	0.000
		No	123				
BMPC (%)		≤30%	90	1.421	0.233	2.790	0.095
		>30%	63				
CPCs		Yes	18	4.545	0.033	9.286	0.002
		No	108				
Myelofibrosis		0-1	101	5.242	0.022	2.532	0.112
		2	33				
Immunophenotyping	CD19	positive	14	0.298	0.585	0.016	0.899
		negative	100				
	CD27	positive	24	1.043	0.307	0.079	0.779
		negative	48				
	CD45	positive	29	0.590	0.442	0.027	0.869
		negative	22				
	CD56	positive	87	2.193	0.139	2.585	0.108
		negative	66				
	CD81	positive	32	0.031	0.861	0.036	0.850
		negative	46				
	CD117	positive	34	0.278	0.598	0.063	0.801
		negative	119				
FISH	IgH	positive	45	1.001	0.317	4.281	0.039
		negative	45				
	TP53	positive	11	16.200	0.000	8.208	0.004
		negative	79				
	RB1	positive	40	0.209	0.648	1.522	0.217
		negative	50				
	1q	positive	48	4.275	0.039	1.756	0.185
		negative	42				
Chromosome Karyotype		others	119	3.059	0.080	5.440	0.020
		complex karyotype	16				
Hb (g/L)		≤85	79	3.134	0.077	1.302	0.254
		>85	74				
PLT (×10^9^/L)		≤100	32	10.022	0.002	18.826	0.000
		>100	121				
ALB (g/L)		≤35	88	0.016	0.899	0.020	0.888
		>35	65				
Cr (μmol/L)		≤177	114	1.772	0.183	0.524	0.469
		>177	39				
Ca (mg/L)		≤2.75	126	5.825	0.016	8.850	0.003
		>2.75	27				
LDH (U/L)		≤200	108	9.882	0.002	11.846	0.001
		>200	45				
β_2_-MG (mg/L)		≤5.5	72	7.928	0.005	4.260	0.039
		>5.5	81				
Treatment duration (months)		≤6	89	1.539	0.215	0.794	0.373
		>6	64				
Treatment response		effective	69	8.237	0.004	5.041	0.025
		ineffective	84				
Autologous stem cell transplantation		yes	37	3.469	0.063	2.232	0.135
		no	116				

### 3.3 Multivariate analysis of prognostic factors for PFS and OS

Multivariate Cox regression analysis identified TP53 deletion, platelet count ≤100×10^9^/L, and LDH >200 U/L as independent adverse prognostic factors for PFS. Additionally, EMD, IgH rearrangement, TP53 deletion, platelet count ≤100×10^9^/L, and LDH >200 U/L were confirmed as independent adverse prognostic factors for OS (**Table 3**).

**Table 3:** Multivariate analysis of prognostic factors in 153 patients with NDMM.

Parameters	PFS			OS		
	HR	95% CI	*P*	HR	95% CI	*P*
R-ISS stage III	0.881	0.199–3.899	0.867	1.011	0.161–6.365	0.991
Extramedullary disease	1.941	0.881–4.279	0.100	2.601	1.115–6.066	0.027
IgH rearrangement	1.199	0.622–2.312	0.588	2.538	1.113–5.787	0.027
TP53 deletion	4.169	1.818–9.558	0.001	3.376	1.366–8.346	0.008
1q amplification	1.456	0.692–3.065	0.322	0.946	0.411–2.179	0.896
PLT ≤ 100×10^9^/L	2.292	1.057–4.969	0.036	3.470	1.401–8.599	0.007
Ca > 2.75 mg/L	1.213	0.419–3.509	0.722	1.102	0.342–3.547	0.871
LDH > 200 U/L	3.102	1.536–6.263	0.002	2.687	1.134–6.368	0.025
β_2_-MG > 5.5 mg/L	1.088	0.272–4.351	0.905	1.200	0.223–6.443	0.832

### 3.4 Impact of bortezomib-based versus non-bortezomib-based therapy on prognosis

**Figure 1:**
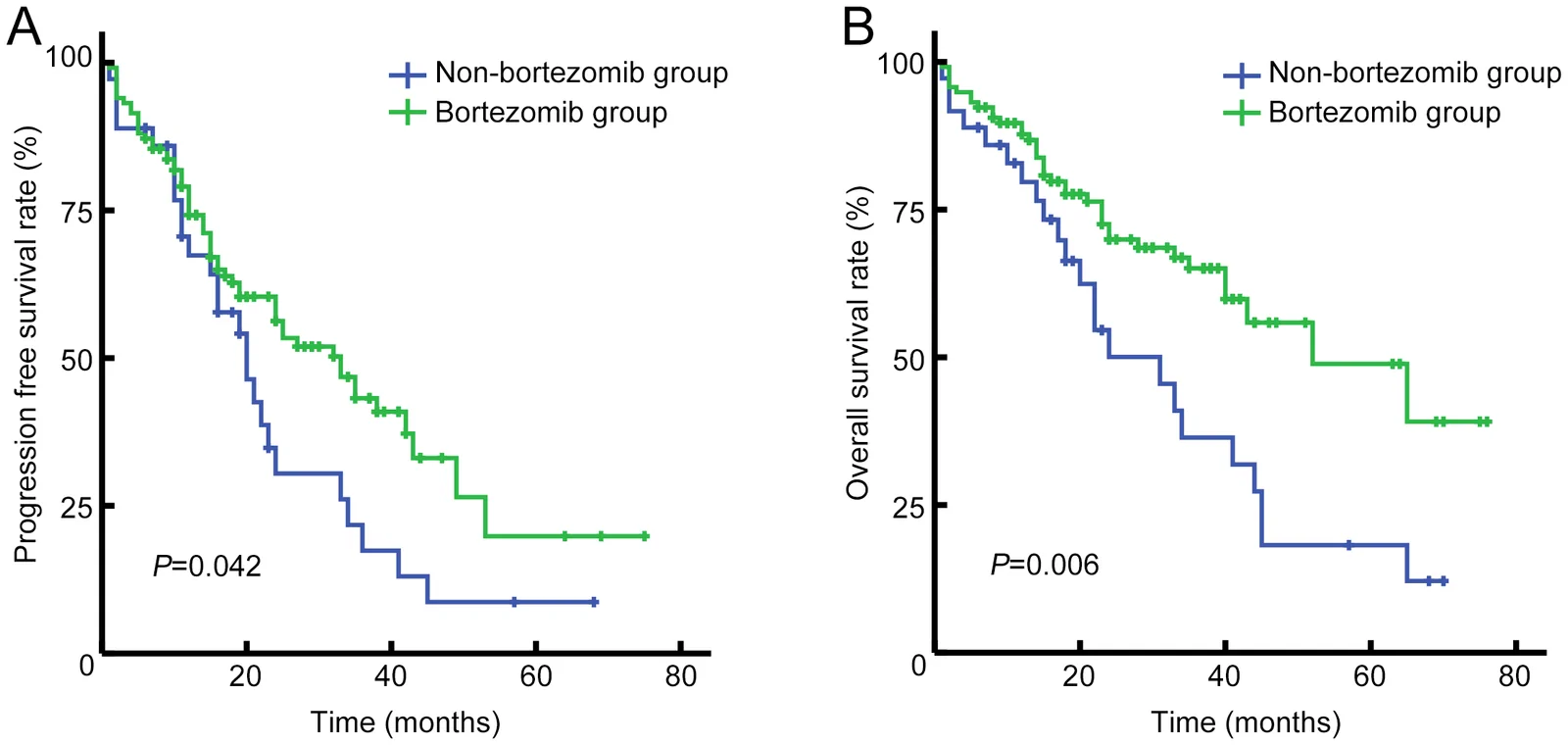
Survival comparisons between patients with NDMM treated with bortezomib-based regimens and non-bortezomib-based chemotherapy. **(A)** The PFS of the bortezomib-based cohort and non-bortezomib cohort. **(B)** The OS of the bortezomib-based cohort and non-bortezomib cohort.

**Figure 2:**
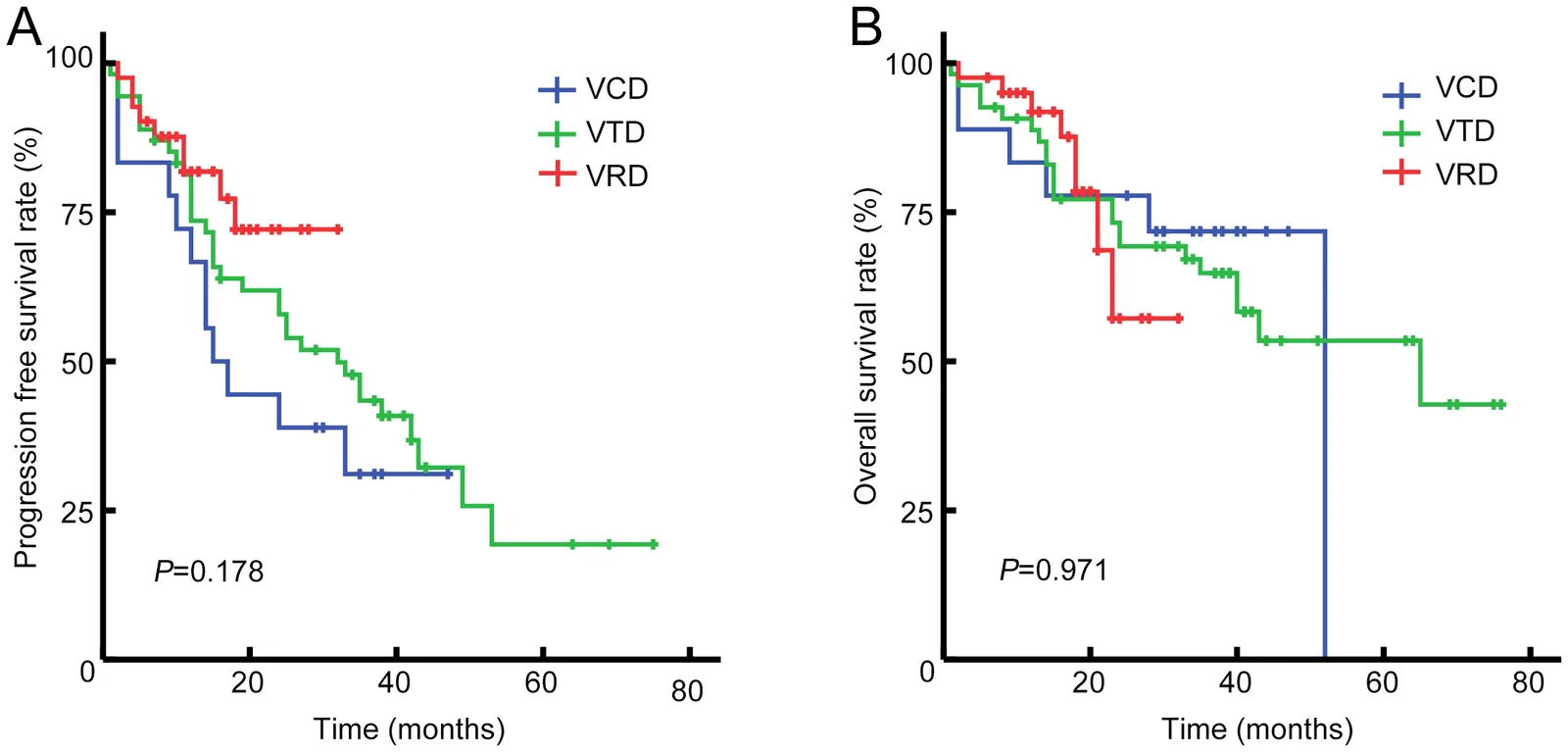
Survival comparisons among patients with NDMM treated with VCD, VTD, and VRD regimens. **(A)** The PFS of the patients treated with VCD, VTD, and VRD regimens. **(B)** The OS of the patients treated with VCD, VTD, and VRD regimens.

Of the 153 NDMM patients, 117 were treated with bortezomib-based chemotherapy and 36 with non-bortezomib-based chemotherapy. The bortezomib-based cohort exhibited significantly longer median PFS and OS compared with the non-bortezomib cohort (median PFS: 33 months vs 20 months, *P* < 0.05; OS: 52 months vs 31 months, *P* < 0.05) (**Figure 1**). No statistically significant differences in median PFS (15 months vs 32 months vs not reached, *P* > 0.05) or OS (52 months vs 65 months vs not reached, *P* > 0.05) were detected among patients treated with VCD (n = 18), VTD (n = 54), and VRD (n = 41) regimens (**Figure 2**).

### 3.5 Impact of bortezomib-based versus non-bortezomib-based therapy on prognosis by CD56 expression status in NDMM

**Figure 3:**
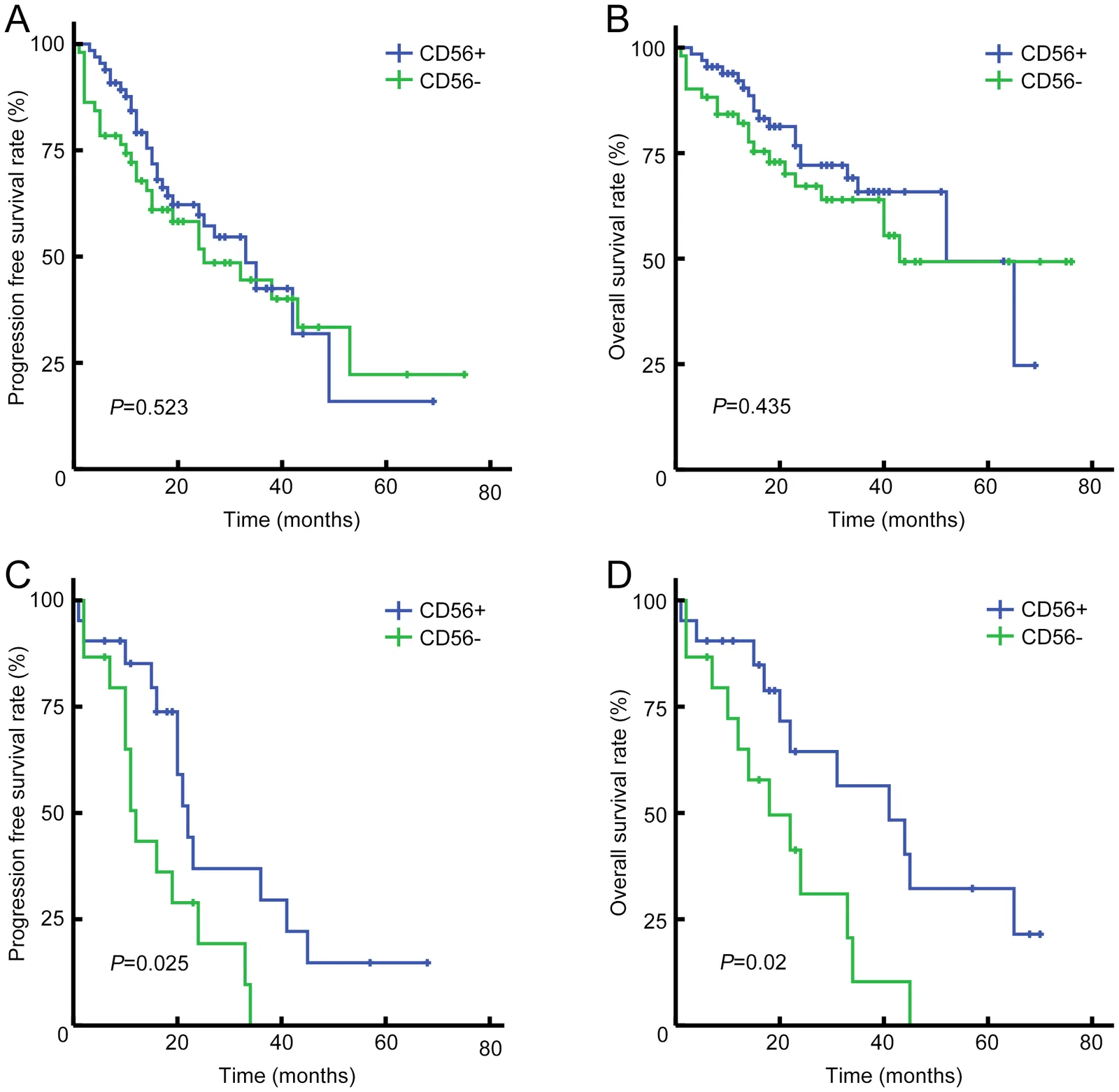
Survival comparisons of NDMM patients stratified by CD56 expression status treated with bortezomib-based regimens or non-bortezomib chemotherapy. **(A, B)** The PFS and OS of the CD56^+^ and CD56^-^ patients in Bortezomib-based cohort. **(C, D)** The PFS and OS of the CD56^+^ and CD56^-^ patients in Non-bortezomib-based cohort.

Among patients in the non-bortezomib-based cohort, CD56^−^ patients (n = 15) had significantly shorter median PFS and OS than CD56^+^ patients (n = 21) (median PFS: 12 months vs 22 months, *P* < 0.05; OS: 18 months vs 41 months, *P* < 0.05). In contrast, within the bortezomib-based cohort, no significant differences in median PFS or OS were observed between CD56^+^ (n = 66) and CD56^−^ (n = 51) patients (median PFS: 33 months vs 25 months, *P* > 0.05; OS: 52 months vs 43 months, *P* > 0.05) (**Figure 3**). Among CD56^−^ patients, no statistically significant differences in median PFS (14 months vs 24 months vs not reached, *P* > 0.05) or OS (NR vs 43 months vs not reached, *P* > 0.05) were noted across VCD, VTD, and VRD treatment subgroups.

### 3.6 Correlation between CD56 expression and clinicopathological characteristics in NDMM patients treated with bortezomib-based therapy

CD56^−^ patients had significantly higher incidences of ISS stage III disease and complex karyotype compared with CD56^+^ patients (ISS stage III: 62.7% vs 42.4%; complex karyotype: 17.8% vs 3.4%, all *P* < 0.05). Additionally, CD56^−^ patients had a higher incidence of thrombocytopenia (29.4% vs 13.6%), elevated β_2_-MG (62.7% vs 42.4%), and MF (33.3% vs 16.7%, all *P* < 0.05). The positivity rate of TP53 deletion was significantly higher in the CD56^−^ group than in the CD56^+^ group (*P* = 0.037). No statistically significant differences were detected between the two groups in other cytogenetic abnormalities (IgH rearrangement: 52% vs 44.9%; RB1 deletion positivity: 48% vs 40.8%; 1q amplification positivity: 56% vs 44.9%, all *P* > 0.05) (**Table 4**).

**Table 4:** Correlation between CD56 expression, clinicopathological features and cytogenetic abnormalities in NDMM patients treated with bortezomib-based therapy [n (%)].

Parameters			CD56^+^ (n=66)	CD56^-^ (n=51)	*χ* *^2^*	*P*
Sex		Male	38 (57.6)	26 (51.0)	0.505	0.477
		Female	28 (42.4)	25 (49.0)		
Age (years)		≤65	45 (68.2)	32 (62.7)	0.378	0.539
		>65	21 (31.8)	19 (37.3)		
DS stage		I+II	16 (24.2)	12 (23.5)	0.008	0.929
		III	50 (75.8)	39 (76.5)		
ISS stage		I+II	38 (57.6)	19 (37.3)	4.755	0.029
		III	28 42.4)	32 (62.7)		
R-ISS stage		I+II	38 (67.9)	18 (50.0)	2.934	0.087
		III	18 (32.1)	18 (50.0)		
M-protein type		IgG	25 (37.9)	19 (37.3)	4.847	0.089
		IgA	26 (39.4)	12 (23.5)		
		others	15 (22.7)	20 (39.2)		
Extramedullary disease		yes	11 (16.7)	14 (27.5)	1.991	0.158
		no	55 (83.3)	37 (72.5)		
BMPC		≤30%	36 (54.5)	32 (62.7)	0.795	0.373
		>30%	30 (45.5)	19 (37.3)		
CPCs		yes	8 (13.6)	7 (17.1)	0.234	0.628
		no	51 (86.4)	34 (82.9)		
Myelofibrosis		0-1	50 (83.3)	30 (66.7)	3.938	0.047
		2	10 (16.7)	15 (33.3)		
Chromosome Karyotype		Others	56 (96.6)	37 (82.2)	5.935	0.015
		complex karyotype	2 (3.4)	8 (17.8)		
FISH	IgH rearrangement	positive	22 (44.9)	13 (52)	0.335	0.563
		negative	27 (55.1)	12 (48)		
	TP53 deletion	positive	4 (8.2)	7 (28)	5.147	0.037
		negative	45 (91.8)	18 (72)		
	RB1 deletion	positive	20 (40.8)	12 (48.0)	0.348	0.555
		negative	29 (59.2)	13 (52.0)		
	1q amplification	positive	22 (44.9)	14 (56.0)	0.366	0.463
		negative	27 (55.1)	11 (44.0)		
Hb (g/L)		≤85	31 (47.0)	26 (51.0)	0.185	0.667
		>85	35 (53.0)	25 (49.0)		
PLT (×10^9^/L)		≤100	9 (13.6)	15 (29.4)	4.391	0.036
		>100	57 (86.4)	36 (70.6)		
ALB (g/L)		≤35	39 (59.1)	25 (49.0)	1.178	0.278
		>35	27 (40.9)	26 (51.0)		
Cr (μmol/L)		≤177	52 (78.8)	35 (68.6)	1.558	0.212
		>177	14 (21.2)	16 (31.4)		
Ca (mg/L)		≤2.75	55 (83.3)	44 (86.3)	0.191	0.662
		>2.75	11 (16.7)	7 (13.7)		
LDH (U/L)		≤200	52 (78.8)	34 (66.7)	2.170	0.141
		>200	14 (21.2)	17 (33.3)		
β_2_-MG (mg/L)		≤5.5	38 (57.6)	19 (37.3)	4.755	0.029
		>5.5	28 (42.4)	32 (62.7)		

## 4 Discussion

Multiple myeloma (MM) arises from the proliferation of clonal plasma cells and primarily manifests as anemia, renal impairment, hypercalcemia, and osteolytic lesions (1, 20). Genetic abnormalities, extramedullary lesions, and elevated lactate dehydrogenase levels indicate a poor prognosis for MM (21–23). Under conventional treatment regimens, MM has a high recurrence rate, with a 5-year survival rate below 50% (24–26). Consistent with the well-established survival benefit of bortezomib (27–29), patients treated with conventional chemotherapy had a median OS of only 31 months, which was significantly inferior to that of patients receiving bortezomib-based regimens.

Currently, bortezomib-based induction regimens combined with immunomodulatory drugs and/or cytotoxic agents are widely used for the management of MM. Commonly employed triplet regimens include VCD, VTD, and VRD. Compared with the VCD regimen, VRD may yield deeper responses, although its impact on long-term survival remains controversial. One study reported a significantly higher 18-month OS rate in patients treated with VRD versus those receiving VCD (30). Another study found no significant differences in 2-year PFS or OS between patients treated with VTD and those treated with VCD (31). In the present cohort, no statistically significant differences in median PFS or OS were observed among NDMM patients treated with the three bortezomib-containing regimens.

The prognostic significance of CD56 in MM remains controversial. CD56 mediates adhesive interactions between myeloma cells and the bone marrow microenvironment; lack of CD56 expression may reduce cellular adhesion, thereby facilitating extramedullary spread and peripheral blood infiltration (32). Prior studies have linked CD56 negativity to adverse clinical features, including extramedullary disease, renal impairment, and plasma cell leukemia (13, 33), although its prognostic impact remains inconsistent across reports (34–36). In the present cohort, CD56 expression status did not influence survival outcomes among patients receiving bortezomib-based therapy, consistent with recent evidence (37). These findings suggest that bortezomib-based regimens may mitigate the adverse prognosis associated with CD56 negativity.

The ISS and R-ISS staging systems are widely used for prognostic stratification in MM. The present study demonstrated that CD56 negativity was significantly associated with ISS stage III disease, in line with previous observations (13, 35). Published data indicate that elevated β_2_-MG and thrombocytopenia are more common in CD56^−^ patients, albeit without statistical significance in prior cohorts (38). In contrast, our analysis revealed significantly higher rates of both abnormalities in the CD56^−^ group. Additionally, CD56 expression status was not significantly correlated with age, sex, or anemia, consistent with prior investigations (32, 39).

Regarding disease dissemination, the relationship between CD56 expression and extramedullary disease (EMD) remains unclear. In our cohort, the incidence of EMD was higher in CD56^−^ patients than in CD56^+^ patients (27.5% vs. 16.7%), although the difference did not reach statistical significance. The presence of CPCs in peripheral blood typically indicates high disease aggressiveness and correlates with advanced ISS stage and inferior OS (40). Although prior studies reported a negative correlation between CPCs and CD56 expression (41, 42), this association was not confirmed in the present cohort, possibly due to the limited number of CPC-positive patients.

In terms of cytogenetic profiles, the frequency of TP53 deletion was significantly higher in the CD56^−^ group (*P* < 0.05). By contrast, IgH rearrangement, RB1 deletion, and 1q amplification were more prevalent in CD56^−^ patients without reaching statistical significance (43, 44). Hundemer et al. reported an association between lack of CD56 expression and t(11;14) translocation (45). TP53 deletion is a well-established independent adverse prognostic factor for both PFS and OS (46, 47), and was detected in 12.2% of our NDMM cohort. These findings suggest that CD56 negativity may reflect a high-risk cytogenetic profile, although cytogenetic analysis was available for only 58.8% of patients.

Notably, CD56 expression has been associated with fibrosis severity in several fibrotic disorders (48). However, the precise pathological mechanisms linking aberrant CD56 expression to myelofibrosis (MF) remain poorly defined. It is hypothesized that CD56 may modulate cellular adhesion, thereby influencing myeloma cell homing and distribution (49). The present study identified MF grade 2 as an adverse prognostic factor for PFS, and CD56 negativity was associated with increased MF severity.

Despite these findings, there are still limitations. This single-center retrospective analysis involved a relatively small sample size of 153 patients. The non-bortezomib cohort was particularly small (n = 36), and further divided into CD56-positive (n = 21) and CD56-negative (n = 15), with insufficient sample size possibly reducing statistical power. The long enrollment period may introduce treatment and care biases, but we found no significant differences in baseline characteristics by comparing different treatment groups. Additionally, data on treatment response, autologous stem cell transplantation (ASCT), maintenance therapy, and subsequent interventions were incomplete, especially in earlier years, which may affect overall survival (OS) outcomes and need to be validated in larger, prospective, multi-center studies.

In summary, CD56 negativity in patients with NDMM is significantly associated with multiple high-risk clinical and cytogenetic features, supporting its role as a correlative marker for aggressive disease. However, in the era of bortezomib-based therapy, CD56 expression status is no longer an independent prognostic factor. This study supports integrating CD56 assessment into initial risk stratification, although its prognostic value should be interpreted in the context of treatment strategy. Large-scale prospective studies with extended follow-up are warranted to establish CD56 as a biomarker for therapeutic stratification.

## Data Availability

The datasets of this study are available from the corresponding author on reasonable request.
